# Hexahydrocurcumin protects against cerebral ischemia/reperfusion injury, attenuates inflammation, and improves antioxidant defenses in a rat stroke model

**DOI:** 10.1371/journal.pone.0189211

**Published:** 2017-12-08

**Authors:** Piyawadee Wicha, Jiraporn Tocharus, Adchara Janyou, Jinatta Jittiwat, Chatchawan Changtam, Apichart Suksamrarn, Chainarong Tocharus

**Affiliations:** 1 Department of Anatomy, Faculty of Medicine, Chiang Mai University, Chiang Mai, Thailand; 2 Department of Physiology, Faculty of Medicine, Chiang Mai University, Chiang Mai, Thailand; 3 Faculty of Medicine, Mahasarakham University, MahaSarakham, Thailand; 4 Division of Physical Science, Faculty of Science and Technology, Huachiew Chalermprakiet University, Samutprakarn, Thailand; 5 Department of Chemistry and Center of Excellence for Innovation in Chemistry, Faculty of Science, Ramkhamhaeng University, Bangkok, Thailand; 6 Center for Research and Development of Natural Products for Health, Chiang Mai University, Chiang Mai, Thailand; Albany Medical College, UNITED STATES

## Abstract

The purpose of the present experiment was to investigate whether hexahydrocurcumin (HHC) attenuates brain damage and improves functional outcome via the activation of antioxidative activities, anti-inflammation, and anti-apoptosis following cerebral ischemia/reperfusion (I/R). In this study, rats with cerebral I/R injury were induced by a transient middle cerebral artery occlusion (MCAO) for 2 h, followed by reperfusion. The male Wistar rats were randomly divided into five groups, including the sham-operated, vehicle-treated, 10 mg/kg HHC-treated, 20 mg/kg HHC-treated, and 40 mg/kg HHC-treated I/R groups. The animals were immediately injected with HHC by an intraperitoneal administration at the onset of cerebral reperfusion. After 24 h of reperfusion, the rats were tested for neurological deficits, and the pathology of the brain was studied by 2,3,5-triphenyltetrazolium chloride (TTC) staining, hematoxylin and eosin (H&E) staining, and terminal deoxynucleotidyltransferase UTP nick end labeling (TUNEL) staining. In addition, the brain tissues were prepared for protein extraction for Western blot analysis, a malondialdehyde (MDA) assay, a nitric oxide (NO) assay, a superoxide dismutase (SOD) assay, a glutathione (GSH) assay, and a glutathione peroxidase (GSH-Px) assay. The data revealed that the neurological deficit scores and the infarct volume were significantly reduced in the HHC-treated rats at all doses compared to the vehicle group. Treatment with HHC significantly attenuated oxidative stress and inflammation, with a decreased level of MDA and NO and a decreased expression of NF-κB (p65) and cyclooxygenase-2 (COX-2) in the I/R rats. HHC also evidently increased Nrf2 (nucleus) protein expression, heme oxygenase-1 (HO-1) protein expression, the antioxidative enzymes, and the superoxide dismutase (SOD) activity. Moreover, the HHC treatment also significantly decreased apoptosis, with a decrease in Bax and cleaved caspase-3 and an increase in Bcl-X_L_, which was in accordance with a decrease in the apoptotic neuronal cells. Therefore, the HHC treatment protects the brain from cerebral I/R injury by diminishing oxidative stress, inflammation, and apoptosis. The antioxidant properties of HHC may play an important role in improving functional outcomes and may offer significant neuroprotection against I/R damage.

## Introduction

Stroke is one of the leading causes of mortality worldwide and is a major cause of long-term disability in people in both developed and developing countries [1, 2]. Middle cerebral artery occlusion (MCAO) is the most common cause of ischemic stroke and still results in high death rates of 40% to 80% [3]. Ischemic stroke causes a reduction in blood flow that is sufficient to alter normal cellular function. Reperfusion is critical in the treatment of ischemic stroke. However, the incidence of post-reperfusion pronounced lesion oxidation occurs because of large amounts of reactive oxygen species (ROS), leading to apoptosis and an inflammatory response, which are frequently associated with a blood brain barrier (BBB) disruption, followed by brain edema [4, 5].

Following cerebral ischemia/reperfusion (I/R), inflammation causes the infiltration of peripheral inflammatory cells, the activation of microglia, and the over-generation of inflammatory mediators, such as cytokines, chemokines, and matrix metalloproteases (MMPs), which are tightly modulated by nuclear factor-kB (NF-κB). Upon an inflammatory response, the cells maintain redox homeostasis by regulating oxidative stress through the induction of phase II antioxidant enzymes, such as heme oxygenase-1 (HO-1), NAD(P)H quinone oxidoreductase-1 (NQO-1), **γ-**glutamylcysteine synthase (**γ-**GCLC), superoxide dismutase (SOD), and catalase (CAT), which are regulated by the nuclear factor erythroid 2-related factor-2 (Nrf2) signaling [6, 7].

The main pathological characteristics of cerebral I/R involve oxidative stress, inflammation, and apoptosis, and these are significantly associated with BBB breakdown, followed by brain edema. Therefore, agents that exhibit antioxidant, anti-apoptotic, and anti-inflammatory properties might be beneficial for the treatment of cerebral I/R. In the present study, our interest was in hexahydrocurcumin (HHC), which is one of the major metabolites of curcumin. The pharmacological activities of curcumin have a wide range of beneficial effects, including anti-inflammatory, antiviral, antioxidant, anticancer, and neuroprotective properties [8–14]. Moreover, several studies report that curcumin also effectively reduces ischemic brain damage. The bioavailability of curcumin is evaluated to be approximately 1%, but 99% of curcumin is represent as glucuronide/sulfate conjugates in blood plasma [15]. Previous studies show that curcumin (dose 0.1 g/kg, intraperitoneal) shows a trace amount in brain tissue (0.4 *μ*g/g) 1 h after dosing [16]. Although curcumin is safe and has many beneficial effects, it is chemically unstable, has poor aqueous solubility, is poorly absorbed in the gastrointestinal (GI) tract, and has a rapid metabolism in blood circulation. Thus, our interest in HHC is due to its higher bioavailability and chemical stability compared to curcumin [17–19]. In this study, we were interested in investigating whether HHC attenuates oxidative stress, inflammation, and apoptosis following cerebral I/R.

## Materials and methods

### Preparation of HHC from curcumin

Curcumin was obtained from *Curcuma longa* L. as described previously [20]. HHC was prepared from curcumin by the method previously described [14]. Briefly, the catalytic hydrogenation of curcumin in ethanol, with palladium on charcoal as a catalyst, followed by the separation of the product HHC from tetrahydrocurcumin and octahydrocurcumin by silica column chromatography, followed by recrystallization with dichloromethane-*n*-hexane, gave a 45% yield of HHC as a white amorphous solid, m.p. 81–82°C. The spectroscopic (IR, ^1^H-NMR and mass spectra) data of the synthesized HHC were identical with those in a previous study [20].

### Experimental animals

Male Wistar rats (weighing 250–300 g) were used for the present study. The animals were obtained from the National Laboratory Animal Center, Mahidol University, Salaya, Nakornpathom, Thailand and were kept under the restriction of a controlled temperature (25±1°C) and light cycles (12 h light/12 h dark). They had free access to food and water. The animal experiments were approved by the Animal Ethics Committee in accordance with the guidelines for the care and use of laboratory animals, as prepared by the Faculty of Medicine, Chiang Mai University Institutional Animal Care and Use Committee. The 170 animals were randomly divided into five groups as follows: (1) sham group (n = 27); (2) vehicle group (n = 46); (3) HHC 10 mg/kg BW (n = 28); (4) HHC 20 mg/kg BW (n = 27); and (5) HHC 40 mg/kg BW (n = 42). HHC was dissolved in 0.1% dimethyl sulfoxide (DMSO) in 1% hydroxylethyl cellulose. The animals (except the sham and the vehicle group) were immediately injected with HHC by an intraperitoneal administration at the onset of cerebral reperfusion. The rats in the vehicle group were injected intraperitoneally 0.1% DMSO in 1% hydroxyethyl cellulose.

### Focal cerebral ischemia

Focal cerebral ischemia was induced via middle cerebral artery (MCA) occlusion by the intraluminal technique [21]. Briefly, the rats were deep anesthetized by an intraperitoneal injection with Zoletil (30 mg/kg) and Xylazine (10 mg/kg). After the rats were completely unconscious, they were place in the supine position on a heating pad for controlling their body temperature, which was in the range of 37.0±0.5°C and were fixed with an adhesive tape. The animals were incised in the midline of the neck and the soft tissues were retracted. The right common carotid artery (CCA) was identified, and this was followed by advancing toward the rostral, which bifurcated into the external carotid artery (ECA) and the internal carotid artery (ICA). An intraluminal filament (Doccol Corporation, Sharon, USA) was introduced past the ECA stump into the ICA until a slight resistance was felt. At this moment, the filament was used to block the origin of the right MCA. After 2 h, the filament was withdrawn carefully to allow MCA reperfusion. Successful occlusion was confirmed by monitoring the cerebral blood flow (CBF) by laser Doppler flowmetry (ADInstruments, Dunedin, New Zealand) in the ipsilateral cortex. The tip of the probe was fixed by an adhesive agent on the intact skull over the ischemic cortex (1 mm posterior and 6 mm lateral from the bregma) [22]. The animals that died or did not show a CBF reduction of at least 70% after ischemia induction were excluded from the study. After the surgery, the animals were transferred to their home cage at 25±1°C and were allowed access to food and water for recovery. The rats were observed closely for the first 2 hours, hourly for the next 6 hours, and four times a day thereafter. The following rats were excluded from our analysis: the rats that died before sacrificing or the rat brains had a subarachnoid hemorrhage or an intraparenchymal hemorrhage. Of the 170 rats that underwent I/R surgery, 5 rats (2.94%) were excluded because of death prior to sacrifice. The mortality rates were as follows: 0% in the sham operated group; 2.35% in the MCAO+vehicle treatment group; and 0.59% in MCAO+10 mg/kg HHC treatment group.

### Neurological evaluation

After the 24-h reperfusion, 6 rats of each group were evaluated for neurological deficits by the method of Longa and coworkers (1989) [21]. The five-point scale was as follows:

Grade 0 = no neurological deficits;
Grade 1 = failure to extend the contralateral forepaw fully when held by the tail;
Grade 2 = circling to the ipsilateral side;
Grade 3 = falling to the contralateral side of brain damage.
Grade 4 = did not walk spontaneously and has depressed level of consciousness.

### Brain and sample preparation

After the experiments were completed (24 h after reperfusion), the rats were humanely sacrificed with an overdose of sodium pentobarbital (100 mg/kg body weight, intraperitoneal) and were intracardially administered an isotonic sodium chloride solution. Then, the brain was removed quickly and frozen at -80°C. The ischemic penumbra was determined according to the method described previously by Ashwa and coworkers [23]. Briefly, the rat brain was placed into the brain slicer matrix and was subsequently cut into three segments beginning 3 mm from the anterior apex of the frontal lobe. The front and back slices were sectioned into slices of 3 mm thickness. The middle slice was coronally cut into slices of 4 mm thickness, which was sectioned longitudinally in the ischemic hemisphere 2 mm from the midline. A transverse diagonal cut was made at the 2 o’clock position to isolate the core from the penumbra. The cerebral penumbra was collected for the biochemical assay and the Western blot. The others were fixed in 4% paraformaldehyde overnight for H&E and TUNEL staining.

### Determination of the infarct volume

At 24 h after the surgery, 6 rats from each group were sacrificed, and the whole brains were rapidly removed. In addition, the infarct volume in the rats treated with 40 mg/kg HHC and the vehicle group was estimated at 2, 4, 6, 12 and 48 h after reperfusion. The brain samples were coronally sectioned into 2-mm-thick slices and were then stained with standard 2% 2,3,5-triphenyltetrazolium chloride (TTC; Sigma-Aldrich Co.) for 15 min at 37°C. Then, the coronal slices were immersed in 4% paraformaldehyde overnight. After TTC staining, the coronal slices were photographed with a digital camera and were analyzed by an image analysis program (ImageJ^®^ software) to determine the extent of the infarct zone. The infarct volume was calculated with the formula: infarct volume (%) = (contralateral hemisphere area–healthy area of ipsilateral hemisphere) × thickness of slice [24].

### Histopathology

At 24 h after reperfusion, 6 rats from each group were anesthetized and perfused intracardially with an isotonic sodium chloride solution, followed by 4% paraformaldehyde in 0.1 M sodium phosphate buffer (pH = 7.4). The brain samples were immediately removed and fixed for 48 h in 4% paraformaldehyde. After that, the brain samples were embedded in paraffin, and the coronal sections were sliced (4 μm thick) and stained with hematoxylin and eosin for observation under a light microscope (Olympus BX51; Olympus Co., Tokyo, Japan).

### Malondialdehyde (MDA) assay

The lipid peroxidation product malondialdehyde (MDA) is used as an indicator of oxidation. The MDA levels were determined via the thiobarbituric acid reaction in the tissue homogenate. Briefly, the rat brains (n = 6 each group) were removed after the 24-h reperfusion. The brains were gently homogenized. The tissue homogenate was incubated with 10% trichloroacetic acid (TCA) and 0.67% (w/v) thiobarbituric acid (TBA) and was heated at 100°C for 30 min. After cooling, the supernatant was transferred into a 96-well plate and read at 532 nm using a microplate reader (BioTek Instruments, Winoaski, VT, USA). The MDA concentration in the brain was determined using a standard curve, and the values are expressed as nmol/mg of tissue protein.

### Griess assay

The NO production was estimated by the Griess reagent method described by Green and coworkers [25]. Briefly, the brain (n = 6 each group) was carefully removed on ice and homogenized in an ice-cold saline solution. The supernatant was allowed to react with 1% sulfanilamide in 5% H_3_PO_4_ and 0.1% N-1-naphthylethylenediamide dihydrochloride in 96-well plates at room temperature in the dark. After 5 min, the supernatant was read at 540 nm using a microplate reader (BioTek Instruments, Winoaski, VT, USA).

### Superoxide dismutase (SOD) assay

This method was used to determine the superoxide dismutase activity by making use of an SOD assay kit (Cayman Company, MI, USA) in which hypoxanthine and xanthine oxidase serve as the superoxide generator, and nitrobluetetrazolium (NBT) is used as the superoxide indicator. Briefly, the rat brains (n = 6 each group) were removed after the 24-h reperfusion. The brains were gently homogenized. The superoxide dismutase (SOD) activity was measured in the supernatant. The changes in the absorbance were observed using a plate reader (BioTek Instruments, Winoaski, VT, USA) at 450 nm. The activity is expressed as U/mg protein.

### Glutathione (GSH) activity

The GSH evaluation was carried out using a GSH assay kit (Cayman Chemical Company, Ann Arbor, MI). After the 24-h reperfusion, the brain (n = 6 each group) was homogenized in a buffer (50 mM phosphate, pH 6–7, containing 1 mM EDTA) and was then centrifuged at 10,000 × g for 15 min at 4°C. A 0.1 mol/l phosphate-buffered saline (pH 7.5) containing 0.6 mmol/l 5,5-dithiobisnitrobenzoic acid (DTNB) and 0.2 mg/ml NADPH was added to the brain supernatant. After mixing, that, the mixture was mixed and glutathione reductase was added to initiate the assay. The absorbance was recorded at 405–414 nm within 15 min. The GSH activities are expressed as μmol/g protein.

### Glutathione peroxidase (GSH-Px) activity

The GSH-Px activity was determined by using a GSH-Px assay kit (Cayman Chemical Company, Ann Arbor, MI). Briefly, the brain samples (n = 6 each group) were homogenized in a buffer (50 mM phosphate, pH 6–7, containing 1 mM EDTA) and were then centrifuged at 10,000× g for 15 min at 4°C. Solutions of 100 μl of phosphate buffer (pH 7.4), 50 μl of co-substrate mixture, 20 μl of cumenehydroperoxide, and 20 μl of the sample were added in the wells, and the absorbance was read once every minute at 340 nm using a microplate reader (DTX800, Beckman Coulter, Austria) to obtain at least five time points.

### TUNEL staining

A TUNEL staining was used to detect cell apoptosis on the basis of DNA fragmentation that results from the apoptosis-signaling cascades. The brain (n = 6 each group) was examined after the 24-h reperfusion with sodium chloride and 4% formaldehyde, regularly embedded in paraffin and was then sectioned at a thickness of 4 μm. TUNEL staining was performed according to the manufacturer’s instructions (Roche Diagnostics Crop., Indianapolis, USA) for the TUNEL assay kit. The total number of cells and the number of TUNEL-positive cells was observed under a light microscope (Olympus BX51; Olympus Co., Tokyo, Japan). Five high-power fields of the ischemic cerebral penumbra areas were randomly selected, and the number of apoptotic cells for each field was counted. The apoptosis index (AI) = the number of positive cells/the number of total cells.

### Western blot analysis

After the 24-h reperfusion, the rat brains were removed and stored at −80°C until use. The brains (n = 3 each group) were gently homogenized in lysis buffer (1.5 mmol/l MgCl_2_, 10 mmol/l KCl, 20 mmol/l HEPES, 1 mmol/l EDTA, 1 mmol/l EGTA, 250 mmol/l sucrose, 0.1 mmol/l phenylmethylsulfonyl fluoride, 1 mmol/l dithiothreitol, and proteinase inhibitor cocktail; pH 7.9). The tissues were centrifuged at 14,000 rpm at 4°C for 15 min. The supernatant was collected and assayed for the determination of the total protein concentrations using the Bradford assay with bovine serum albumin (BSA) as the standard.

The nuclear extraction was performed according to the method described previously by Dignam and coworkers with some modifications [26]. Briefly, the brain tissue was homogenized in an ice-cold hypotonic lysis buffer containing 10 mM HEPES (pH 7.9), 1.5 mM magnesium chloride (MgCl_2_), 10 mM potassium chloride (KCl), 0.5 mM phenylmethylsulfonyl fluoride (PMSF), 0.5 mM dithiothreitol (DTT), a protease inhibitor and 1% NP-40. Then, the homogenate was centrifuged at 13,000 rpm for 30 sec at 4°C. The nuclear pellet was resuspended in an ice-cold hypertonic extraction buffer containing 10 mM HEPES (pH 7.9), 0.42 M NaCl, 1.5 mM MgCl_2_, 10 mM KCl, 0.5 mM PMSF, 1 mM DTT and protease inhibitors at 4°C for 30 min. After centrifugation at 14,000 g for 5 mins, the nuclear extract was collected, and the protein concentrations were determined by using the Bradford protein assay.

Equal amounts of protein were separated on a 10–15% SDS polyacrylamide gel and were then transferred to a PVDF membrane (Immobilon-P, Millipore, Bedford, MA, USA) at 400 mA for 35 min. After blocking with 5% skim milk in TBS containing 0.1% Tween-20 (TBST) at 4°C for 3 h, the membrane was incubated with primary antibodies (anti-Bax, anti-Bcl-X_L_, anti-caspase-3, anti-Nrf2, anti-HO-1, anti-p65, and anti-COX2) at 4°C overnight. Then, the membrane was washed in TBST, which was followed by an incubation with horseradish peroxidase-conjugated secondary antibodies for 1 h at room temperature. The blots were then incubated for 5 min with an ECL substrate before detection of the luminescence band by blue X-ray films. The densitometry was analyzed by using ImageJ^®^ software.

### Statistical analysis

All the values are presented as the mean±S.D. The statistical differences between the two groups were determined by Student’s t-test. Other data were analyzed using a one-way analysis of variance, followed by a post hoc Dunnett’s test, to compare the significance between the individual groups. The differences were significant when the *p*-value was less than 0.05. All the experiments were carried out three times, and the mean value and standard deviation were calculated.

## Results

### Regional cerebral blood flow (rCBF) monitoring during the transient middle cerebral artery occlusion (MCAO) procedure

**Fig 1:**
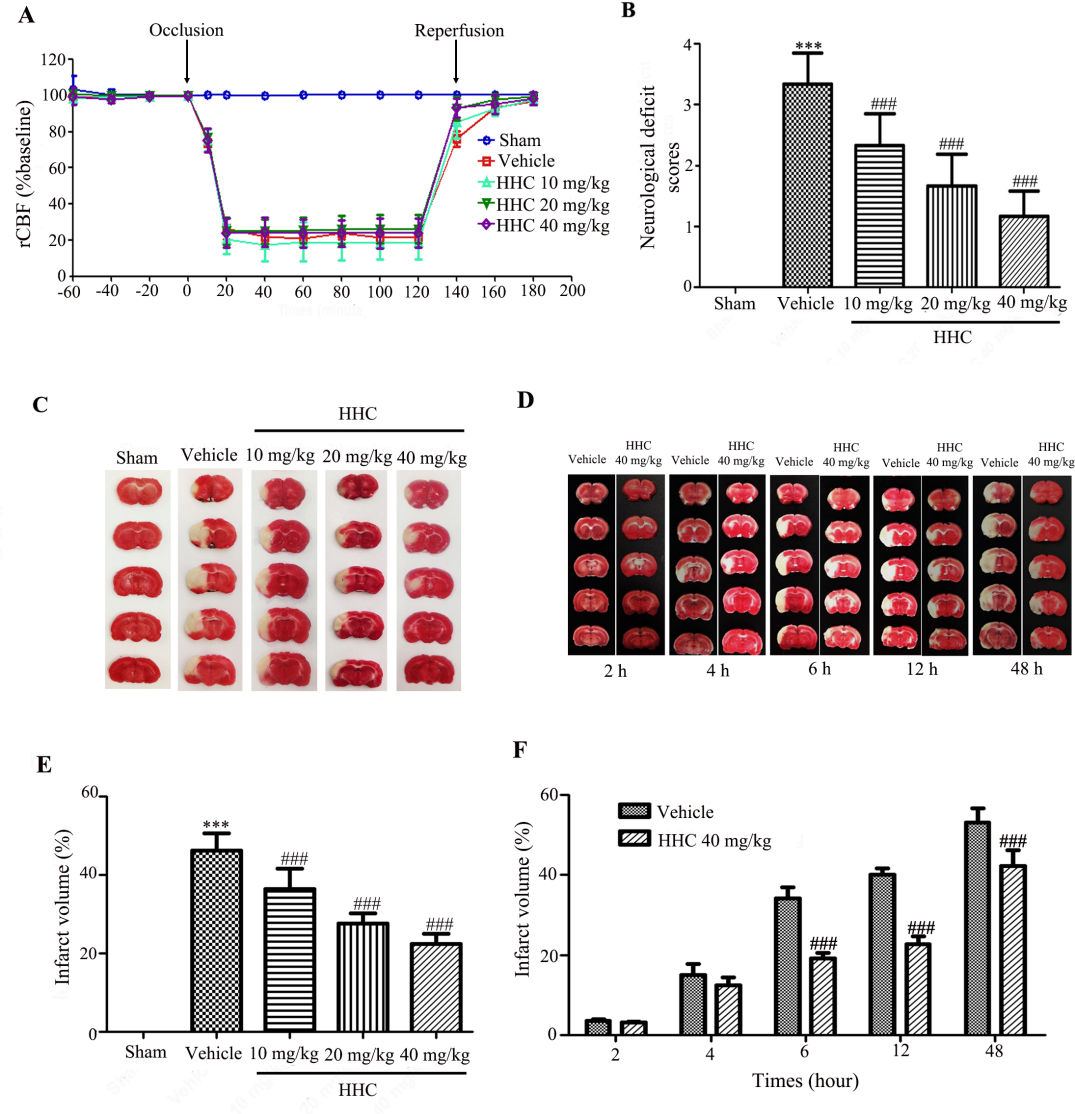
Effects of HHC on brain infarction and neurological outcomes after cerebral I/R in rats. **HHC was administered upon initiation of the reperfusion. (A)** The regional cerebral blood flow (rCBF) observed during pre-occlusion, occlusion, and reperfusion. **(B)** The neurological deficit scores after cerebral I/R. **(C)** The TTC staining of series coronal brain section (2 mm thick) after 24-h reperfusion. The infarct region is in white color, whereas the non-ischemic region appears in red color. **(D)** The TTC staining of rat treatment with 40 mg/kg HHC and vehicle group at 2 h, 4 h, 6 h, 12 h and 48 h after reperfusion. **(E)** The percentage of infarct volume after I/R in different groups. **(F)** The percentage of infarct volume after I/R in various reperfusion time. All the data are presented as mean±SD. *** *P* < 0.001 versus sham group. ^###^ *P* < 0.001 versus vehicle group.

The rCBF was measured during pre-ischemia, ischemia, and reperfusion, as shown in Fig 1A. Before occlusion, there were no significant differences in the CBF between the groups. The CBF showed no significant changes in the sham operation at all times during the operation. The CBF in the I/R rats, which were administered vehicle and HHC at doses of 10 mg/kg, 20 mg/kg, and 40 mg/kg, was observed to be reduced immediately to less than 30% of the baseline after occlusion. During reperfusion, the CBF returned to a level higher than 70% of the baseline after reperfusion in all the I/R groups without any significant differences detected among the groups (*P* > 0.05). This result confirms the success of the animal model.

### HHC attenuates the neurological deficit and infarct volume in stroke rats

To investigate the neuroprotective effect of HHC on I/R injury, we first evaluated the neurological deficit at 24 h after reperfusion, as shown in Fig 1B. The results revealed that the vehicle group had higher neurological deficit scores than the sham group (*P* < 0.001). The neurological deficit scores were significantly reduced in the HHC-treated rats at all doses when compared to the vehicle group (*P* < 0.001).

Cerebral infarction was assessed by TTC staining at 24 h after reperfusion. The infarct areas after I/R are presented in white in the right hemisphere that is shown in Fig 1C. The TTC staining of the brain slices were examined for the percentage of the infarct volume relative to the whole brain. The percentage of the infarct volume significantly increased in the vehicle group when compared with the sham group (Fig 1E). Noticeably, the percentage of the infarct size was significantly attenuated by HHC (10 mg/kg, 20 mg/kg, and 40 mg/kg) when compared with the vehicle group. Moreover, we investigated the infarct volume at 2 h, 4 h, 6 h, 12 h and 48 h after the reperfusion in the MCAO+40 mg/kg HHC treatment group and in the MCAO+vehicle treatment group (Fig 1D). There were no differences in infarct volume at 2 h and 4 h after reperfusion between the MCAO+40 mg/kg HHC treatment group and in the MCAO+vehicle treatment group. The infarct volume was markedly attenuated by 40 mg/kg HHC at 6 h, 12 h and 48 h after reperfusion compared to the vehicle treatment group (*P* < 0.001) (Fig 1F).

### HHC recovers neuronal morphologic damage in stroke rats

**Fig 2:**
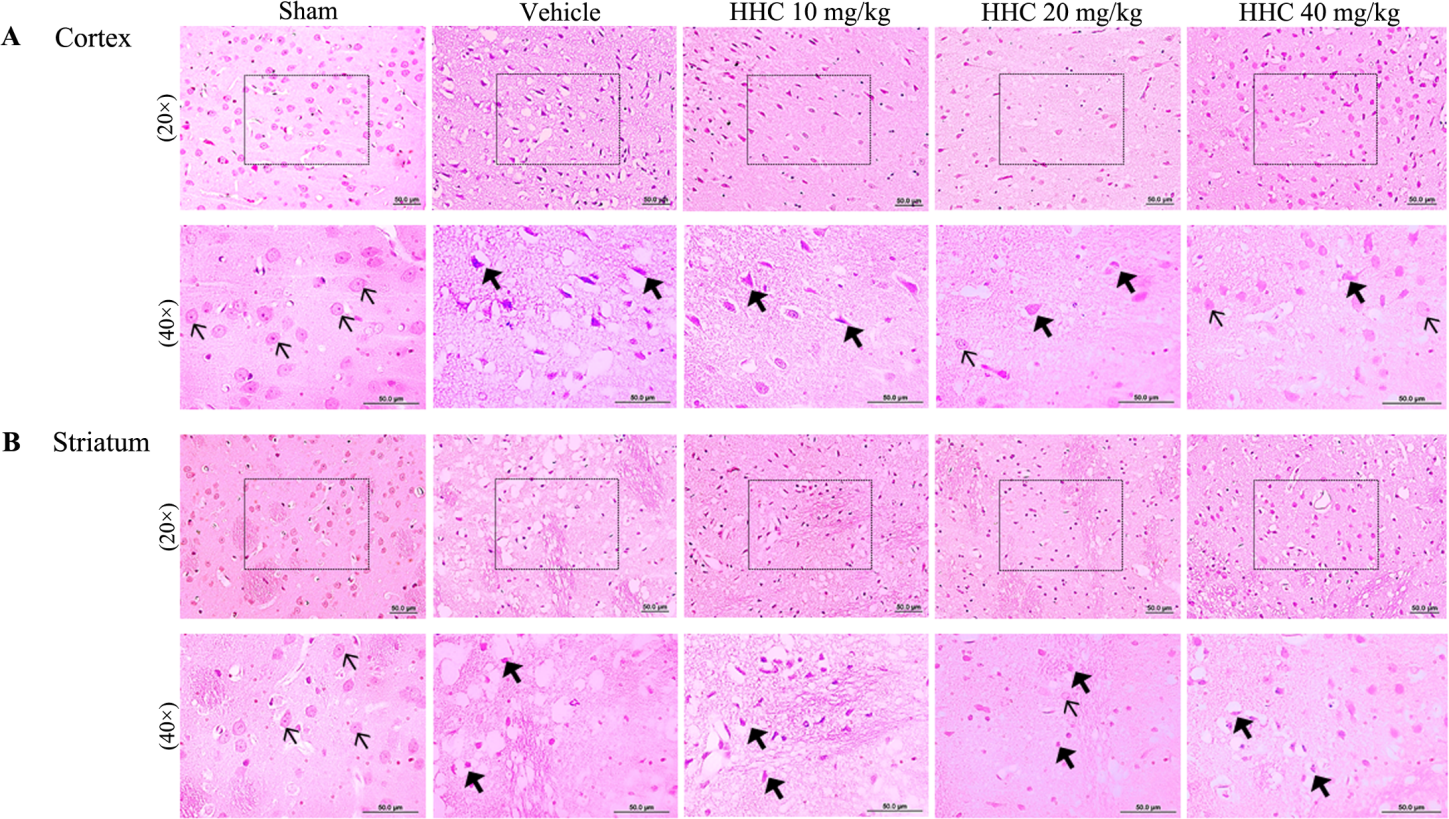
The representative histopathological changes of the brain at 24 h after focal cerebral I/R in stroke rats. The brain tissues were stained with H&E and visualized with a light microscope (20× and 40×). In the sham group, the structure of most of the neurons in the cortex and the striatum were clear (thin arrow). The neurons of the I/R group were found to present shrinkage, chromosome condensation, and nuclear pyknosis, and showed increased intercellular space (thick arrow). The number of intact neurons in the rats that were treated with HHC was found to have increased.

The brain infarction caused by I/R was observed in the ipsilateral brain slices stained with H&E. The results indicate that the sham group had normal staining in both the cortex and the striatum (Fig 2). The brain sections of the sham group remained intact and with normal cell organelles, the neurons were still arranged well, and the nuclei were centered with clear staining, whereas the vehicle group showed many vacuolated spaces and neuronal loss. The brain of the HHC treatment groups (10 mg/kg, 20 mg/kg, and 40 mg/kg) decreased the number of degenerated neurons, and the number of normal neurons increased in the ischemic penumbra cortex.

### HHC reduces lipid peroxidation in stroke rats

**Fig 3:**
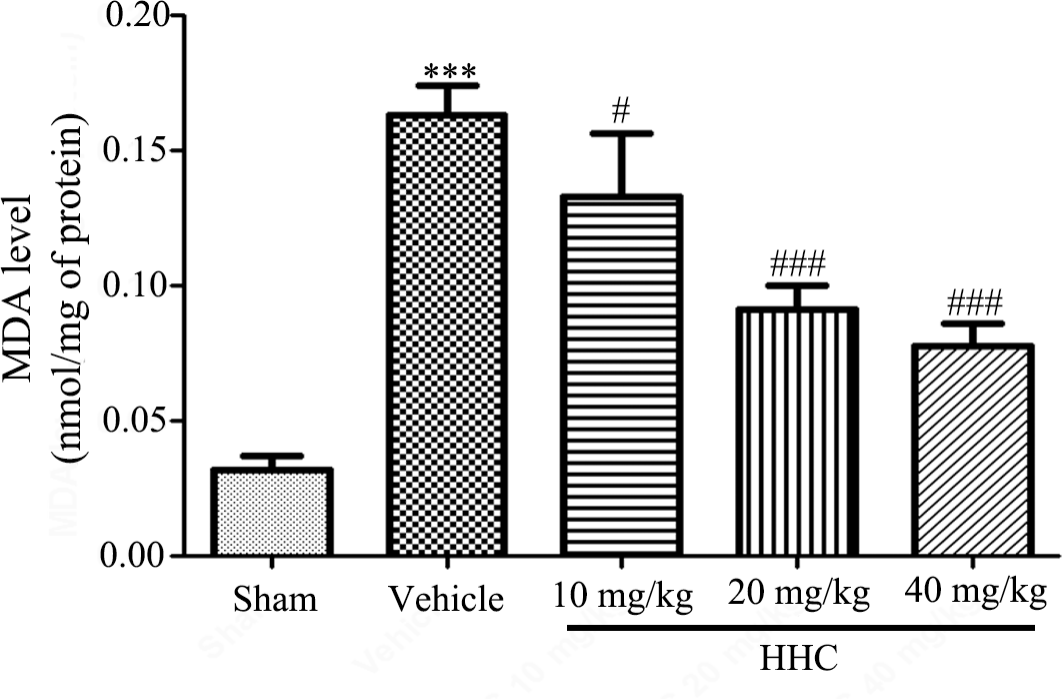
HHC reduces markers of lipid peroxidation in the ischemic tissue. The effects of HHC on the MDA level in different groups. The data are presented as mean±SD (n = 6). *** *P* < 0.001versus sham group. ^#^ *P* < 0.05 versus vehicle group, and ^###^ *P* < 0.001 versus vehicle group.

The lipid peroxidation after the I/R damage were demonstrated by the MDA levels in the brain. The data revealed that a significant elevation in the MDA levels in the brain was present in the vehicle group compared to the sham group (*P* < 0.001) (Fig 3). HHC, at doses of 20 mg/kg and 40 mg/kg, produced a significant reduction in the MDA (*P* < 0.001) levels compared with the vehicle group.

### HHC reduces inflammation and improves antioxidant defenses in the tissue

**Fig 4:**
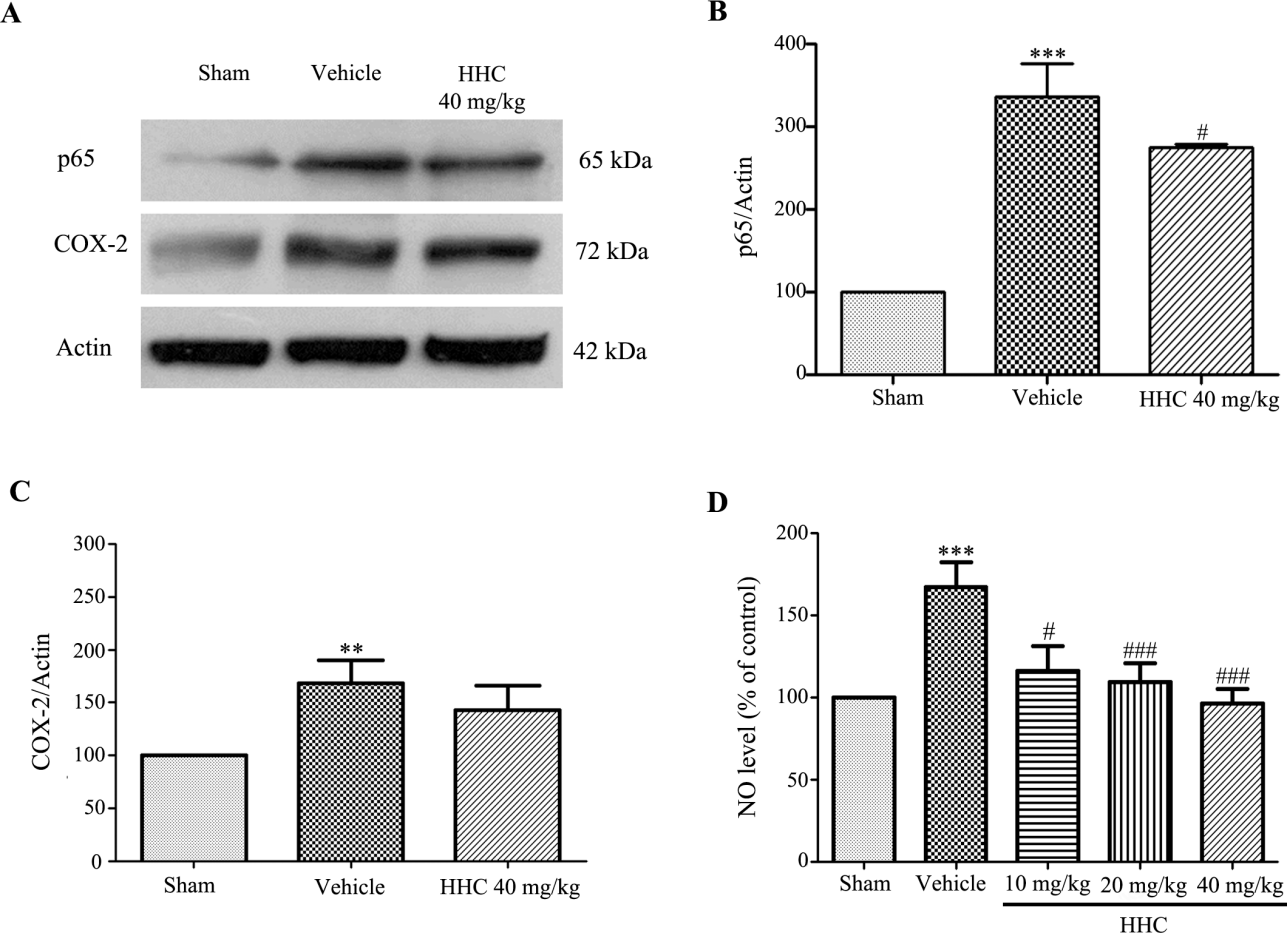
The effect of HHC on inflammation in cerebral I/R rats. **(A)** Representative of the NF-κB (p65) and the COX-2 protein expressions, as examined by western blot analysis. **(B)** The quantitative results of the expressions of the NF-κB (p65) proteins in each of the groups. **(C)** The quantitative results of the expressions of the COX-2 proteins in each of the groups. **(D)** The effect of HHC on the NO level in stroke rats. The data are expressed as mean±SD. ** *P* < 0.01 versus sham group, and *** *P* < 0.001 versus sham group. ^#^ *P* < 0.05 versus vehicle group, and ^###^ *P* < 0.001 versus vehicle group.

The NF-κB (p65) expression in the ischemic penumbra cortex was assessed by a Western blot assay. As shown in Fig 4A and 4B, the NF-κB (p65) expression in the vehicle group was markedly higher than the NF-κB (p65) expression in the sham group (*P* < 0.001); moreover, HHC at a dose of 40 mg/kg significantly prevent the increasing of the NF-κB (p65) expression (*P* < 0.05). COX-2 also increased in the vehicle group compared to the sham group (*P* < 0.001), as shown in Fig 4A and 4C. However, HHC, at a dose of 40 mg/kg, slightly reduced the expression of COX-2. Moreover, we determined the NO levels in the brain, which were estimated by the Griess reagent method. The analysis revealed that the NO levels significantly increased in the vehicle group compared with the sham group (*P* < 0.001) (Fig 4D). Evidently, the HHC treatment attenuated the NO levels dose-dependently.

**Fig 5:**
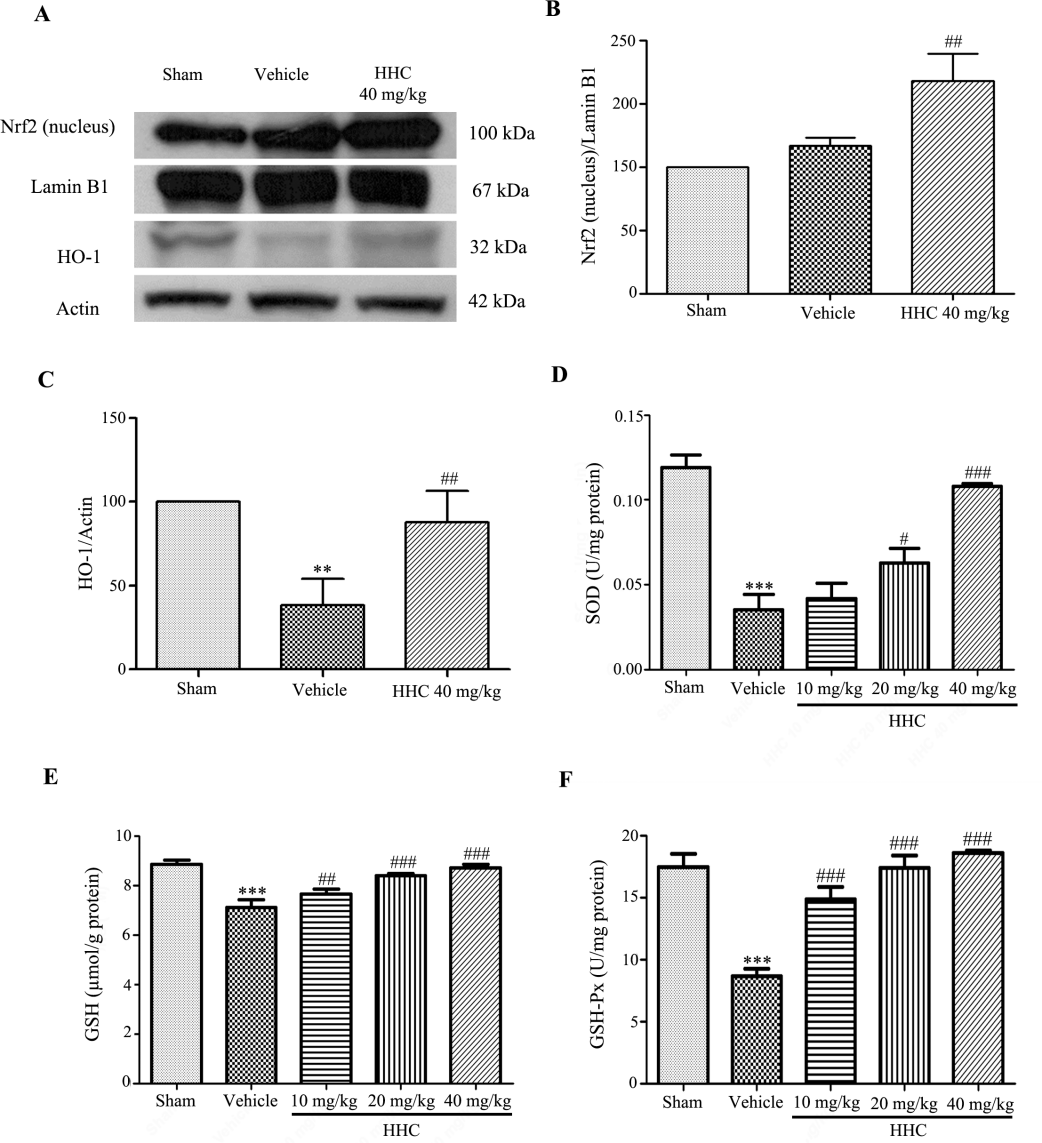
The effect of HHC on the Nrf2 signaling pathway in cerebral I/R injury rats. **(A)** Representative of the Nrf2 (nucleus) and the HO-1 protein expressions, as examined by western blotting. **(B)** The quantitative results of the expressions of the Nrf2 proteins in each of the groups. **(C)** The quantitative results of the expressions of the HO-1 proteins in each of the groups. **(D)** The effect of HHC on SOD activity in cerebral I/R injury rats. **(E)** The effect of HHC on GSH in cerebral I/R injury rats. **(F)** The effect of HHC on GSH-Px in cerebral I/R injury rats. The data are presented as mean±SD. ** *P* < 0.01 versus sham group, and *** *P* < 0.001 versus sham group. ^#^ *P* < 0.05 versus vehicle group, ^##^ *P* < 0.01 versus vehicle group, and ^###^ *P* < 0.001 versus vehicle group.

To evaluate the neuroprotective effect of HHC on oxidative damage in the I/R rats, we examined the nuclear translocation of Nrf2 by a Western blotting. In addition, the protein expression of HO-1 and the antioxidant enzyme activities, such as SOD, GSH, and GSH-Px, were determined. The data revealed that the rats that were treated with HHC had significantly increased Nrf2 protein expression in the nucleus (*P* < 0.01), as shown in Fig 5A and 5B. These data confirm the neuroprotective effect of HHC to initiate an adaptive cell response via the antioxidant pathway. Next, we further examined the function of HHC with regard to the antioxidative defense mechanism by determining the expression of HO-1, a phase II antioxidant enzyme gene that is related to the transcription of Nrf2. As shown in Fig 5A and 5C, the expression of the HO-1 protein was significantly reduced after MCAO, whereas HHC at a dose of 40 mg/kg prevents the reduction of the HO-1 expression (*P* < 0.01). Moreover, treatment with HHC significantly increased the levels of SOD, GSH, and GSH-Px (Fig 5D, 5E and 5F, respectively). The findings indicate that HHC improves the antioxidant properties via increasing of the expression and the activity of the Nrf2 signaling pathway, which activates antioxidant proteins and antioxidant enzyme activity in stroke rats.

### HHC treatment reduces apoptotic markers in the ischemic tissue

**Fig 6:**
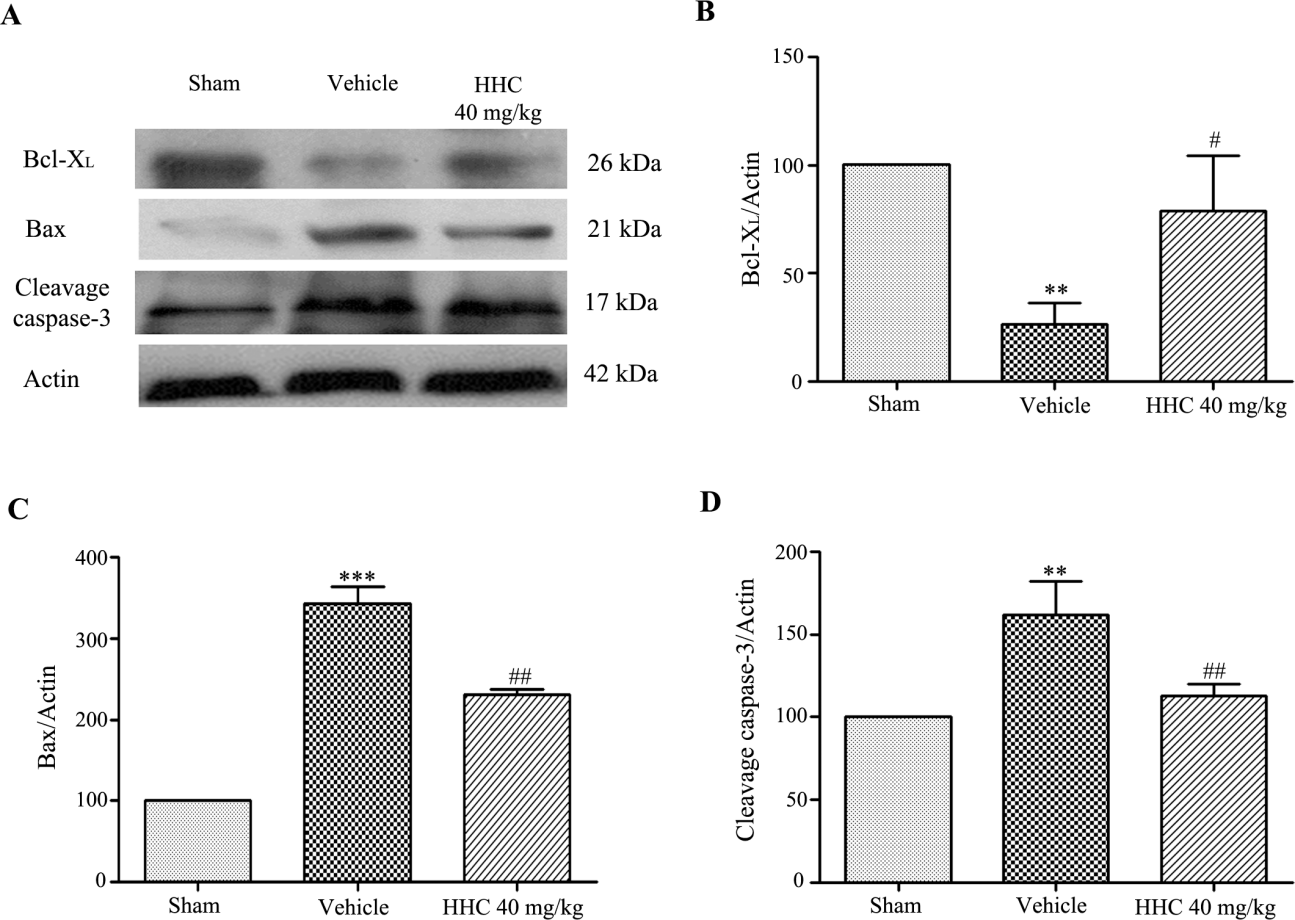
The effect of HHC on the apoptosis pathway in stroke rats. **(A)** Representative of the Bax, the Bcl-X_L_, and the cleaved caspase-3 protein expressions in the penumbra cortex, as determined by western blot analysis. **(B)** The quantitative results of the expressions of the Bcl-X_L_ proteins in each of the groups. **(C)** The quantitative results of the expressions of the Bax protein in each of the groups. **(D)** The quantitative results of the expressions of the cleaved caspase-3 protein in each of the groups. The data are presented as mean±SD. ** *P* < 0.01 versus sham group, and *** *P* < 0.001 versus sham group. ^#^ *P* < 0.05 versus vehicle group and ^##^ *P* < 0.01 versus vehicle group.

To clarify whether the neuroprotection of HHC was associated with an anti-apoptosis pathway, the expression of Bcl-X_L_, Bax, and cleaved caspase-3 in the penumbra area were determined by a Western blot analysis. The experiment showed that the expressions of Bax (*P* < 0.001) and cleaved caspase-3 (*P* < 0.01) increased in the vehicle group, whereas the expression of Bcl-X_L_ in the vehicle group was reduced compared with the sham group (*P* < 0.01) (Fig 6). In contrast, the expressions of Bax and cleaved caspase-3 significantly decreased with the HHC treatment, whereas the Bcl-X_L_ protein level increased with HHC treatment at 40 mg/kg in comparison to the vehicle group (*P* < 0.05).

**Fig 7:**
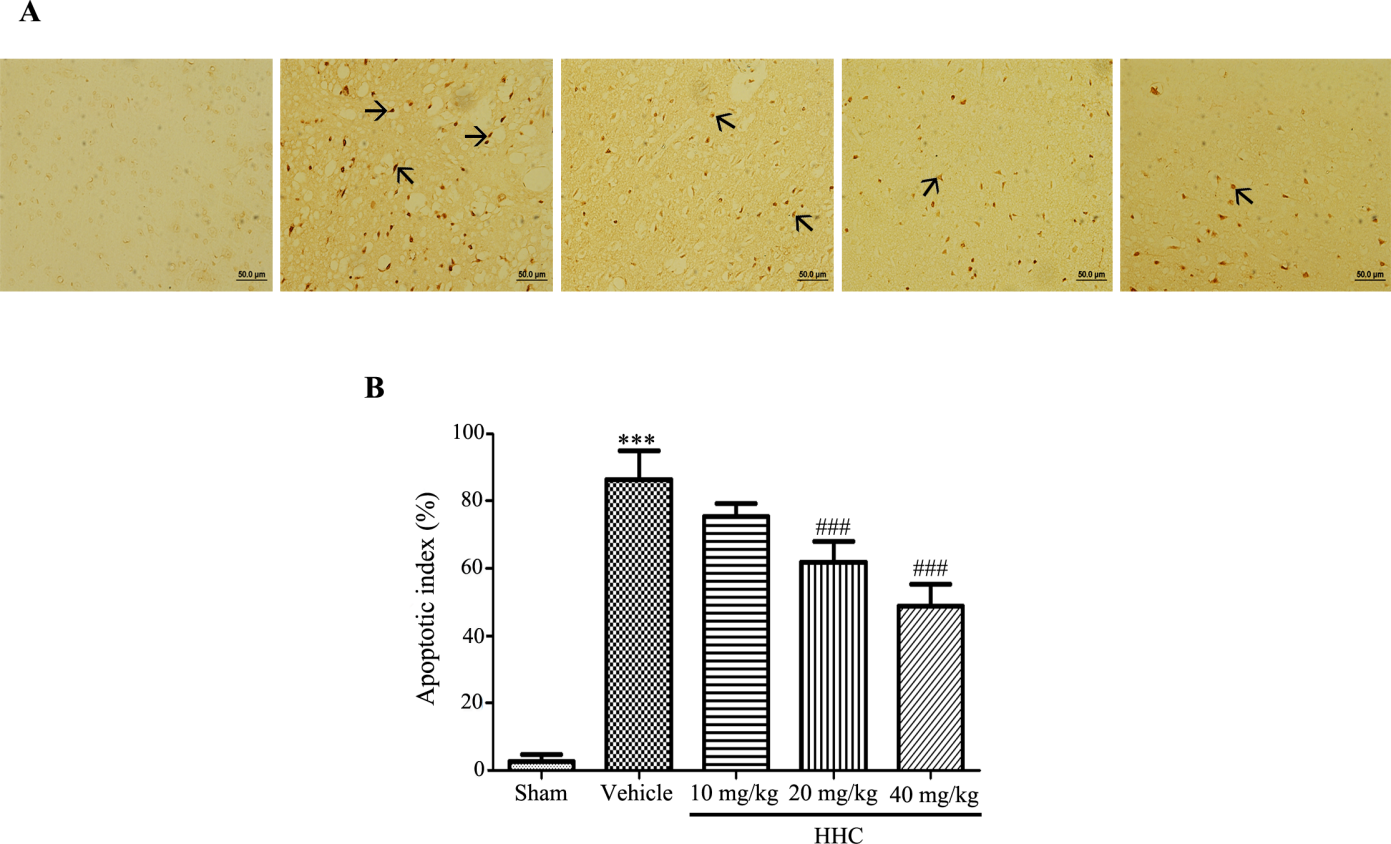
The effect of HHC on I/R-induced neuronal apoptosis. **(A)** The apoptotic cells examined by TUNEL staining and which were visualized with a light microscope (20×). The arrows show TUNEL-positive cells. **(B)** The quantitative analysis of TUNEL-positive cells in each of the groups. The data are presented as mean±SD. ***P < 0.001 versus sham group. ^###^ *P* < 0.001 versus vehicle group.

The investigation to confirm whether HHC reduced apoptosis was carried out using TUNEL staining. TUNEL-positive cells exhibiting shrunken cell bodies and containing numerous apoptotic bodies and darkly stained cells were considered apoptotic cells. At 24 h after reperfusion, a few TUNEL-positive cells were present in the sham group, whereas the number of TUNEL-positive cells observed in the vehicle group was markedly increased in comparison with the sham group (*P* < 0.001). Treatment with HHC at doses of 20 mg/kg and 40 mg/kg showed a significantly attenuated number of TUNEL-positive cells in the ischemic cerebral penumbra areas compared with the vehicle group (Fig 7). These results confirm that HHC shows anti-apoptotic effects in I/R damage.

## Discussion

Ischemic stroke causes a reduction in blood flow that is sufficient to alter the normal cellular function. Oxygen and glucose deprivation during cerebral ischemia triggers a cascade of events, including the disruption of the membrane potential due to the reduction in the ATP production and mitochondrial membrane damage, which leads to a release of excitatory neurotransmitters, such as glutamate [27, 28]. Reperfusion is critical in the treatment of ischemic stroke. However, reperfusion may worsen tissue injury in excess of the injury caused by ischemia alone. Cellular injury after reperfusion of previously viable ischemic tissues is described as I/R injury [29]. I/R injury may occur in reperfusion after thrombolytic therapy in ischemic stroke, and this causes the formation of large amounts of ROS, which results in brain injury via several mechanisms. In clinical practice, I/R injury, which results in brain infarction, is the major cause for worsening of brain dysfunction and neuronal death [30]. There are very few proficient treatments for ischemic stroke, and the evolution of new therapeutics is currently the main goal in many laboratories worldwide [31, 32]. In the present study, we were interested in HHC, a natural metabolite of curcumin, which is produced from phase I of curcumin metabolism. Data from several studies support the therapeutic effects of curcumin, such as its antioxidant, anti-inflammatory, anti-carcinogenesis, antiviral, and anti-fungal effects, in addition to its neuroprotective effects [8–14]. Previous studies report that HHC shows a higher chemical stability as well as has higher bioavailability than the parent curcumin [19]. Additionally, HHC has a higher antioxidant activity than the parent curcumin [17, 18]. Therefore, it is possible that HHC, one of the major metabolites of curcumin, may decrease oxidative stress and inflammation and, thereby, decrease neuronal apoptosis in I/R injury. Moreover, the neuroprotective effect of HHC on cerebral ischemic damage is not yet reported and requires further study. In this study, we first investigated whether HHC attenuates neuronal damage via the activation of antioxidative activities, anti-inflammation, and anti-apoptosis following cerebral I/R.

The incidence of post-reperfusion lesions and oxidative stress refers to elevated intracellular levels of ROS, which may result in damage to tissue, lipids, proteins, and DNA. ROS directly damages cellular membranes through lipid peroxidation [33, 34]. A previous study reported that rats pre-treated with curcumin (100 mg/kg i.p.) for 5 days before MCAO showed markedly decreased lipid peroxidation [35]. Moreover, rats treated with curcumin (300 mg/kg i.p.) at reperfusion exhibited significantly decreased MDA levels [36]. In our present study, rats subjected to I/R presented high levels of MDA in the brain tissue. In contrast, the MDA levels decreased in the HHC-treated group. Moreover, after administration of HHC (10 mg/kg, 20 mg/kg, and 40 mg/kg) attenuated the number of degenerated neurons and increased the number of normal neurons in the ischemic penumbra cortex. These findings suggest that HHC protects from brain damage caused by I/R-induced oxidative stress.

Inflammatory responses are observed in the brain following stroke [37]. ROS activates downstream signaling pathways, including the NF-κB signaling pathway, which plays a role in inflammation [38]. NF-κB is one of the most important transcription factors activated after MCAO. NF-κB is involved in the inflammatory responses that potentiate cerebral ischemic damage, thus activating many genes involved in the pathogenesis of cerebral ischemia, such as iNOS, IL-1β, TNF-α, ICAM-1, COX-2, and IL-6 [39–42]. The overexpression of iNOS and COX-2 is an important determinant of ischemic stroke, which leads to the progression of brain injury [43]. Previous studies report that cerebral ischemia models indicate that NF-κB’s actions are widely harmful and that animals treated with a pharmacological NF-κB inhibition exhibit efficient anti-inflammatory effects [44, 45]. Many studies have also recommended that inhibiting NF-κB activation brings about the prevention and helps develop smaller brain infarctions [44, 46, 47]. Curcumin exhibits an anti-inflammatory effect on oxygen glucose deprivation (OGD)-injured brain microvascular endothelial cells (BMECs) through NF-κB signaling pathway [48]. Furthermore, curcumin (200 mg/kg) attenuates inflammation by decreasing inflammatory mediators, such as interleukin-1 beta (IL-1β), tumor necrosis factor-alpha (TNF-α), Prostaglandin E2 (PGE2), NO, COX-2, and iNOS, induced by brain ischemia in rats [49]. Our results found that HHC at a dose of 40 mg/kg significantly decreased the expressions of NF-κB (p65) and reduced the NO level in the brain compared to the vehicle group. Moreover, the percentage of brain infarct volume was attenuated in the HHC-treated group. These data suggest that the diminution of the cerebral ischemic volume may be partly because of inflammation.

Upon an inflammatory response, cells maintain redox homeostasis by regulating oxidative stress through the induction of phase II antioxidant enzymes, such as HO-1, NQO-1, and **γ-**GCLC, which are regulated by Nrf2 signaling. The activation of Nrf2 signaling induces the translocation of Nrf2 into the nucleus and mediates its transcriptional activity, resulting in the increased expression of antioxidant enzymes that play important roles in scavenging free radicals, such as SOD and CAT [6, 7]. A previous study found that the deficiency of Nrf2 is associated with the addition of inflammatory cytokine production in a brain injury model [50]. Furthermore, NF-κB activation that is induced by lipopolysaccharide (LPS) is ameliorated by Nrf2 activators, such as allyl isothiocyanate (AITC), sulforaphane (SUL), and curcumin [51]. Therefore, Nrf2 signaling and its related activities may play an important role in the defense against oxidative stress possibly by the activation of antioxidant activities as well as the elimination of inflammatory pathways. Previous studies report that curcumin (100 mg/kg i.p.) up-regulated Nrf2 and HO-1 expression and increased endogenous antioxidant defense enzymes in the MCAO model [52]. This result showed that the treatment with HHC suppressed the loss of the proteins Nrf2 and the HO-1 and the SOD and GSH-Px activity, and the GSH levels that returned toward the sham levels after I/R. Moreover, Nrf2 (nucleus) was significantly increased under the ischemic reperfusion condition and was further enhanced by HHC. These findings suggest that HHC prevents the reduction of endogenous antioxidant enzymes activities, consequently increasing the protective defense mechanisms through the antioxidant pathway. This finding is consistent with the findings of previous studies, which demonstrate that curcumin protects against the cerebral brain damage caused by cerebral I/R. This effect may occur through the up-regulation of the transcription factor Nrf2 expression, which may be one of the strategic targets for cerebral I/R therapies. Thus, it is probable that HHC is a factor inducing antioxidant activation and, accordingly, leads to the decrease in the ROS, oxidative stress, and inflammation caused by cerebral I/R injury.

Moreover, the excessive production of ROS induces apoptosis. Many studies demonstrate that apoptosis plays an important role in cerebral ischemic pathogenesis. Many previous studies successful show that apoptosis leads to progress in brain infarction with DNA fragmentation. Experimental evidence indicates that caspase-3 knock-out mice and Bcl-2-overexpression both decrease ischemic infarction after MCAO [53, 54]. Therefore, the ideal preventive or therapeutic approach would indeed target apoptosis after cerebral I/R. Here, we presented the down-regulation of Bax and cleaved caspase-3 and the up-regulation of Bcl-X_L_ in the ischemic brain after treatment with HHC. Additionally, this study also showed a small number of TUNEL-positive cells in the ischemic brain of the HHC-treated group, clearly indicating the anti-apoptotic effect of HHC on cerebral I/R injury. This finding is consistent with the findings of previous studies, which demonstrate that curcumin (300 mg/kg i.p.) significantly decreases the expression of caspase-3 protein and TUNEL-positive cells in transient cerebral ischemic reperfusion [55]. From all our results, it is evident that HHC treatment protects the brain of stroke rats from cerebral I/R injury by diminishing oxidative stress and inflammation and that HHC has antioxidant properties, which might play a role in improving functional outcomes and might offer significant neuroprotection against I/R damage.

In conclusion, our study in animal models indicates that the effect of HHC in transient cerebral I/R injury might be that it protects the brain from damage and improves neurological outcomes by attenuating oxidative stress, inflammation, and apoptosis and activating endogenous antioxidant defenses.

## Supporting information

S1 File Data of rCBF during MCAO, neurological deficit score, infarct volume, lipid peroxidation, expression of inflammation and antioxidant proteins, nitric oxide levels, SOD levels, GSH levels, GSH-Px levels and apoptotic index. (XLSX)
